# Intraspecific *ITS* Variability in the Kingdom *Fungi* as Expressed in the International Sequence Databases and Its Implications for Molecular Species Identification

**DOI:** 10.4137/ebo.s653

**Published:** 2008-05-26

**Authors:** R. Henrik Nilsson, Erik Kristiansson, Martin Ryberg, Nils Hallenberg, Karl-Henrik Larsson

**Affiliations:** 1 Department of Plant and Environmental Sciences, University of Gothenburg, Box 461, 405 30 Göteborg, Sweden; 2 Mathematical Statistics, Chalmers University of Technology/Department of Zoology, University of Gothenburg, 412 96 Göteborg, Sweden

**Keywords:** fungi, molecular species delimitation, intraspecific variation

## Abstract

The internal transcribed spacer (*ITS*) region of the nuclear ribosomal repeat unit is the most popular locus for species identification and subgeneric phylogenetic inference in sequence-based mycological research. The region is known to show certain variability even within species, although its intraspecific variability is often held to be limited and clearly separated from interspecific variability. The existence of such a divide between intra- and interspecific variability is implicitly assumed by automated approaches to species identification, but whether intraspecific variability indeed is negligible within the fungal kingdom remains contentious. The present study estimates the intraspecific *ITS* variability in all fungi presently available to the mycological community through the international sequence databases. Substantial differences were found within the kingdom, and the results are not easily correlated to the taxonomic affiliation or nutritional mode of the taxa considered. No single unifying yet stringent upper limit for intraspecific variability, such as the canonical 3% threshold, appears to be applicable with the desired outcome throughout the fungi. Our results caution against simplified approaches to automated *ITS*-based species delimitation and reiterate the need for taxonomic expertise in the translation of sequence data into species names.

## Introduction

That DNA sequence information is assigned material importance in contemporary mycology is exemplified by the recent notion of fungal barcoding, which seeks to standardize DNA-borne species identification through the use of one or more gene sequences from aptly chosen and annotated reference specimens (Hebert et al. 2003; Seifert et al. 2007; Bruns et al. 2008). The need for such protocols in mycology is patently clear: the vast number of extant fungi coupled with the dwindling number of taxonomic experts and the recondite nature of fungal life jointly make a persuasive case for barcoding-type approaches to species identification in the fungi (Guarro et al. 1999; Taylor et al. 2000; Schadt et al. 2003). The most popular locus for DNA-based mycological studies at the subgeneric level, and hence for species identification, is the internal transcribed spacer (*ITS*) region of the nuclear ribosomal repeat unit (Horton and Bruns, 2001; Bridge et al. 2005). This multi-copy, tripartite, and roughly 550-basepair (bp) segment combines the advantages of resolution at various scales (*ITS1*: rapidly evolving, *5.8S*: very conserved, *ITS2*: moderately rapid to rapid; Hillis and Dixon, 1991; Hershkovitz and Lewis, 1996) with the ease of amplification of a multi-copy region into a readily obtainable product whose variability typically reflects synapomorphies at the species level.

Genome scans and novel molecular insights have brought attention to other genes of various copy number—notably the mitochondrial cytochrome C oxidase I (*COI*; Hebert et al. 2003; Little and Stevenson, 2007; Seifert et al. 2007)—that potentially could meet the occasional shortcomings of the multi-copy *ITS* region, such as pleomorphism and alignment difficulties (c.f. Álvarez and Wendel, 2003; Avis et al. 2006; Feliner and Rosselló, 2007). While the use of these new regions for purposes of species identification is certain to complement—perhaps even replace—that of the *ITS* region in some groups of fungi (Geiser et al. 2004), the difficulty associated with their generic primer design and amplification from low-quantity samples such as herbarium specimens suggest that the much more easily amplified ribosomal DNA will remain in frequent use for the foreseeable future (Tautz et al. 2003; Bruns and Shefferson, 2004; Blaxter et al. 2005). Many aspects of the nuclear ribosomal repeat region are but partly understood, however, and the prospects of the region as a barcode for the fungi has mainly been evaluated within limited taxonomic scopes. Using all 4185 available, fully identified fungal species represented by at least two satisfactory *ITS* sequences in the International Nucleotide Sequence Database (INSD: GenBank, EMBL, DDBJ; Benson et al. 2007), this study pursues the following questions:

### Can intraspecific *ITS* variability in the fungi be captured in one generally applicable yet stringent interval, such as 0–3%?

It is often assumed, implicitly or otherwise, that fungal intraspecific variability is comparatively low and generally applicable across the kingdom such that it can be represented by a percentage interval, notably 0–3% (c.f. Cohan, 2002; Izzo et al. 2005; Ciardo et al. 2006). While this indeed seems to be the case for some groups of fungi (Druzhinina et al. 2005; Hinrikson et al. 2005; Smith et al. 2007), such a contention probably does not hold true for others (Martin et al. 2002; Edwards and Turco, 2005). As the absence of such a fungal-wide interval would be expected to compromise automated attempts at separation of intra- and interspecific variation, it would be of value to attain detailed knowledge on intraspecific *ITS* variability in all fungi presently available to the mycological community through INSD.

### Is *ITS1* always more variable than *ITS2*?

Much attention has been focused on *ITS1* as the more variable sublocus of the two and thereby, presumably, the better species marker (Chen et al. 2001; Narutaki et al. 2002; Hinrikson et al. 2005). There are, however, observations to the contrary (Leaw et al. 2006), and knowledge of the extent of this deviance, as well as of any systematic signal in it, would add to our understanding of how *ITS*-based species identification efforts best be designed.

### Is the *ITS* a straightforward barcode region for the fungi?

The *ITS* region is more often advocated than cautioned against as a vector for species identification in fungi, but these reports are typically based on subsets of fungi, often at the family level or lower (Sugita et al. 1999; Henry et al. 2000; Iwen et al. 2002). The picture emerging from joint analysis of all available fungal *ITS* sequences should be highly indicative of the performance of the *ITS* as a barcode region in the fungi.

This study uses the INSD data on an as-is basis. It is well known that the taxonomic reliability in public sequence databases is less than ideal (Bridge et al. 2003; Binder et al. 2005; Nilsson et al. 2006) and that there are other compounding factors such as chimeric sequences and obsolete classification systems and synonyms (Ashelford et al. 2005; Bidartondo et al. 2008; Ryberg et al. 2008) that render difficult the extrication of true taxonomic signal from the welter of noise surrounding it. While this will always hamper automated approaches to *en masse* sequence analysis, the present study takes several measures to account for these complications as to provide reasonably objective answers to the above questions.

## Materials and Methods

### Compilation of data

All fungal *ITS* sequences identified to species level in INSD as of August 6, 2007 were downloaded using *emerencia* 1.0 (Nilsson et al. 2005). The 2995 entries identified by Nilsson et al. (2006) as potentially misidentified or otherwise problematic were discarded from the study. Similarly, sequences with less than 100 bp. in either of *ITS1* or *ITS2*, as well as sequences with more than 1% IUPAC DNA ambiguity symbols in any of the three *ITS* subregions, were excluded. Hidden Markov Models (HMMs) of the flanking nuclear small sub-unit (*nSSU*), *5.8S*, and nuclear large sub-unit (*nLSU*) were constructed from the large-scope fungal alignments of Tehler et al. (2003); Larsson et al. (2004); Binder et al. (2005); and James et al. (2006) using HMMER 2.3.2 (Eddy, 1998). After calibration, the HMMs enabled *in silico* extraction of *ITS1*, *5.8S*, and *ITS2* from the downloaded sequences using Perl (Supplementary Document 1).

### Data analysis

Intraspecific pairwise alignments of all loci considered (*ITS1*, *5.8S*, *ITS2*, and jointly) were generated in Clustal W 1.83 (Thompson et al. 1997) for all 4185 species for which satisfactory INSD data from two or more specimens were available. Sequence similarity in the form of absolute, uncorrected (Hamming) distances (c.f. Minichini and Sciarrino, 2006) for all combinations of two conspecific specimens were computed in Python (Supplementary Document 1); from these distance matrices, median intraspecific similarities for each species were retrieved as to further reduce the impact of potentially contestable records using the statistical language R 2.5.1 (R Development Core Team, 2007; Supplementary Document 1). For the 16 species represented by more than 100 *ITS* sequences in INSD, the estimates were based on a random sample of 100 sequences from these. To derive global values for the intraspecific variability of the kingdom *Fungi* and its five conceptual phyla (*Ascomycota, Basidiomycota, Chytridiomycota*, *Glomeromycota,* and *Zygomycota*), weighted averages with weights proportional to the number of available sequences for each species were computed; the weighting scheme employed assigns higher importance to well-sampled species without disregarding more poorly represented species.

## Results and Discussion

The ease with which the *ITS* region can be amplified from a variety of fungi in various morphs and states of preservation—as well as its high level of synapomorphic variability in many groups of fungi—have given impetus to several *ITS*-based barcoding-type efforts for select groups of fungi (e.g. Druzhinina et al. 2005; Kopchinskiy et al. 2005; Kõljalg et al. 2005). Given the decisive role assigned to the region, it may perhaps seem curious that many of its facets remain poorly understood, and the present study seeks to provide the data needed to examine these in a critical way. The questions pursued and the results obtained are constrained by, and to some extent reflective of, the wanting state of taxonomic reliability in the public sequence databases. Attempts were made to correct for outlier sequences, thereby abating the impact of inconsistent application of species names and the vagaries of laboratory work. Even so, for some common root-and soil associated fungi such as *Rhizoctonia*, Latin binomials seem little more than convenient placeholders under which specimens are subsumed in the absence of conflictory, but also confirmatory, evidence. Our results furthermore capture taxonomic complications such as the hypothesized presence of hybridisation in *Tricholoma sulphureum* (Comandini et al. 2004) and cryptic speciation in *Laetiporus sulphureus* (Rogers et al. 1999). As such the data obtained seem to corroborate one of the corollaries arising from the barcoding debate, namely that it may not lie in the interest of the mycological community to allow open and non-validated submission of barcodes to the international sequence databases. Similarly, continuous curation of taxonomic and nomenclatural aspects of reference sequences on part of both the sequence authors and the database in question appears a crucial element of molecular mycology.

One noticeable aspect of our assessment of fungal intraspecific variability is that the uncertainty of the estimates tends to decrease as the number of conspecific sequences available for any given species increases (Supplementary Document 2). In other words, more than some few conspecific sequences may be required to encompass the genetic variation found among populations of distinct localities. This observation calls into question the considerable number of barcoding studies based on less than a handful of collections per species (c.f. Little and Stevenson, 2007) and indeed the use of a single, defining sequence as arbiter of conspecificity in the first place. Other conveyors of amalgamated information, notably HMMs and multiple alignments (Eddy, 1998; Nilsson et al. 2004), appear much more suited to capture and relay such complexity.

### Intraspecific *ITS* variability

**Figure 1:**
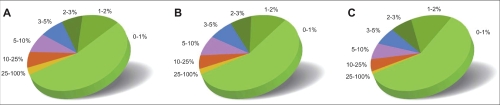
The proportion of fungal species in this study having intraspecific variabilities in the ranges depicted of (**A**) the *ITS1* region, (**B**) the *ITS2* region, and (**C**) the *ITS1*, *5.8S*, and *ITS2* regions combined.

The fungal intraspecific *ITS* variability as expressed in INSD does not readily lend itself to partitioning into clearly defined units. As defined in Materials and Methods, the weighted average of the intraspecific *ITS* variability of the kingdom *Fungi* is 2.51% with a standard deviation (SD) of 4.57 (*Ascomycota*: 1.96%, SD 3.73; *Basidiomycota*: 3.33%, SD 5.62; *Chytridiomycota*: 5.63%, SD 10.49; *Glomeromycota*: 7.46%, SD 4.14; *Zygomycota*: 3.24%, SD 6.12; Table 1). The comparatively well-studied *Dikarya* (*Ascomycota* and *Basidiomycota*) stands out as less variable than the basal fungal lineages, although these regions of the kingdom are rather sparsely represented by *ITS* sequences such that taxonomic intricacies and the deficient state of some sequence data are likely to attain a higher degree of penetration for these taxa. The canonical 3% threshold value for intraspecific variation fares surprisingly well for the fungi (Fig. 1), but it is nevertheless refuted by multiple examples from all fungal phyla (Supplementary Document 3).

Interestingly, our results also offer examples of well-and independently sampled species with low or no intraspecific variability (e.g. *Boletus pinophilus* and *Serpula lacrymans;* Supplementary Document 3). The wide spread in intraspecific variability observed testifies to the apparent futility of trying to find a single unifying yet stringent fungal-wide cut-off value to demarcate intra- from interspecific variability (Fig. 1; Supplementary Document 2). Such divides between intra- and interspecific variability—barcoding gaps—will, if at all in existence, have to be sought in different regions of variation space depending on the taxa under consideration; there is furthermore little to suggest that such divides in similarity could be deduced by taxonomic knowledge and logic alone. Even when collapsing the mushroom-forming *Agaricomycetes* in Supplementary Document 3 into formal orders as applicable (Hibbett et al. 2007), no such group could be classified as “easily barcoded”: all orders feature taxa that are considerably below, roughly at, and markedly above the fungal-wide average for intraspecific *ITS* variability. A similar pattern is observed when grouping these fungi according to putative nutritional mode, but whether these inferences will persist in the light of extended taxon sampling remains at issue.

### Internal *ITS* variability

Our results show that the variability of *ITS1*, at least on average, exceeds that of *ITS2* (Fig. 1). The difference in variability is noticeable at times (e.g. *Hypocrea citrina* and *Malassezia furfur*, both with >3% difference). In other cases, such as *Boletus edulis* and *Cordyceps bassiana—*with less than 1% difference—and *Agaricus bisporus* and *Alternaria brassicae*—with no difference—it is less conspicuous. For 34% of the fungal species compared, however, *ITS2* is more variable than *ITS1*, which refutes the common assumption that *ITS1* always is the most variable spacer of the *ITS* region. Indeed, it would seem likely that for certain taxa, *ITS2* represents a better vector of low-level taxonomic information. We did not find evidence, however, for any phylum-wide systematic component to this observation as comparatively higher levels of *ITS2* variability could not be significantly related to phylum-wise affiliation (Fisher’s exact test, p-value >0.05). The overall variation in *ITS1* and *ITS2* was found to be highly correlated (0.87; Supplementary Document 2) which supports the view that the two regions do not evolve independently of one another.

The *5.8S* is typically fully conserved within a species, and the variation sometimes observed is negligible (weighted average: 0.21%, SD 0.67). Counterintuitively, therefore, the region can be expected to interfere with pre-defined threshold values for intraspecific variation. Supplementary Document 3 shows that even if both the *ITS1* and *ITS2* are more than 3% variable within one and the same species, the inclusion of the very conserved *5.8S* may serve to reduce the apparent variability of the joint region into less than 3%, thereby masking the distinctness indicated by its flanking regions (as is the case for, e.g. *Rhizopogon roseolus* and *Xerocomus subtomentosus*). Our data suggest that the *5.8S*, while arguably useful in other contexts (Hershkovitz and Lewis, 1996; Larsson et al. 2004), may be best left out from such estimates.

### The *ITS* region as a fungal barcode

The ever-increasing prevalence of fungal environmental samples generated in ecological studies accentuates the need for automated, high-throughput approaches to species identification, and many such initiatives are indeed centered around the *ITS* region. This study shows that the *ITS* region is not equally variable in all groups of fungi (Table 2) and that the variation does not seem to be easily correlated to the systematic affiliation or nutritional mode of the species. These disparities speak against automated species delimitation using, for example, a global 3% cut-off value. To devise efficient fungal barcodes based on the *ITS* region will require, it would seem, far-reaching taxonomic knowledge specific to each group of fungi; a large number of conspecific specimens from as many populations and geographical regions as can be reasonably achieved; and possibly the erection of one or more tailored, closed-submission databases for the purpose. Criticism has been raised against the barcoding community for not taking these matters seriously enough (Wheeler, 2004; Will et al. 2005; Meier et al. 2006), and the present study lends further weight to the importance of these claims.

## Conclusions

A plexus of pleomorphic organisms, fungi often defy assignment to genus or even family level, and it is becoming progressively apparent that molecular information will soon take over the role as the primary source for reliable species identification in all but for some few groups of fungi. It is moreover clear that these methods have only begun to reveal the true face of fungal diversity in that the absolute majority of fungi still await discovery and formal description (Hawksworth, 2001; Blackwell et al. 2006; Schmit and Mueller, 2007). Much of this diversity is recovered from ecological samples such as soil and plant debris in total absence of any physical manifestation of the fungi present. The mere observation that the multi-copy *ITS* region can be amplified from these low-quantity samples, whereas many low- and single-copy genes currently cannot, implies that the *ITS* region will remain a mycological cornerstone for a long time to come. That the region typically shows variation within, and to an even larger extent among, species turns the region into a valuable vector for mycological pursuits, although one for which not all preconceived ideas and assumptions hold true. The large number of fungi for which the *ITS* has been generated further serves to increase the usefulness of the region for purposes of comparison, but whether it will ever be truly useful also for automated species delimitation remains an open question—and one that the present results do not seem to answer in the affirmative.

## Supplementary Material

Supplementary document 1 Perl code used for *in silico* extraction of *ITS1*, *5.8S*, and *ITS2* from the fungal *ITS*-region sequences in INSD using HMMs; Python code used for alignment and similarity comparison; and R code used for calculating the statistics. Released under the GNU-GPLv2 software license.

Supplementary document 2 (**a**) A histogram of the number of fungal *ITS* sequences per species as included in this study, showing that the majority of species is represented by fewer than five sequences. (**b**) The number of conspecific sequences plotted against the median intraspecific variability for the species in question, showing a decrease in the uncertainty of the estimates with higher number of sequences. Deviant sequences attain a higher degree of penetration in sparsely sampled species than in more richly sampled ones, where the larger sample sizes lead to estimates of smaller variance. (**c**) The variability of *ITS1* (x axis) plotted against that of *ITS2* (y axis) on a logarithmic scale. The correlation coefficient is 0.87 (p-value <10^−16^). (**d**) A histogram of the number of species in the study with an intraspecific variability in the ranges indexed, showing the asymmetric, long-tailed distribution of intraspecific variability. Jointly with (a), the histogram gives a good overview of the present state of *ITS*-borne sampling of fungi.

Supplementary document 3 Estimated intraspecific variability of all 4185 fungal species of this study; results boiled down to *ITS1*, *5.8S*, *ITS2*, and all combined. The number of sequences underlying the estimates, as well as the phylum-wise affiliation as given in INSD, are indicated. Extreme values are likely, but not necessarily bound, to hint at the presence of cryptic species or other unresolved taxonomic issues, laboratory artefacts, or additional compounding factors and were found to be distributed in all phyla in proportion to their size (Chi^2^ test: p-value >0.2). In the interest of completeness, no such entries were left out from the study. Only organisms annotated in INSD as belonging to the kingdom *Fungi* are included; organisms traditionally treated as “fungal allies” but now known to belong elsewhere were not targeted in this study.

## Figures and Tables

**Table 1:** Statistics on all species and sequences included in this study; data as of August 6, 2007. The standard deviation is shown in brackets as applicable.

Number of included species	4185 (973 genera)
Number of species excluded due to being represented by only one (satisfactory) sequence	5428
Average number of sequences per species	7
Total number of pairwise alignments of the study	~2 million (13 Gb)
Percentage of the estimated 1.5 million fungal species represented by at least two *ITS* sequences	0.28%
Median length of the loci (bp)	183 (*ITS1*), 158 (*5.8S*), 173 (*ITS2*)
Weighted intraspecific *ITS* variability in the kingdom *Fungi*	2.51% [4.57]
Weighted intraspecific *ITS* variability for the five conceptual phyla of the kingdom	
*Ascomycota* (2509 species)	1.96% [3.73]
*Basidiomycota* (1582 species)	3.33% [5.62]
*Chytridiomycota* (11 species)	5.63% [10.49]
*Glomeromycota* (23 species)	7.46% [4.14]
*Zygomycota* (60 species)	3.24% [6.12]
Most abundantly represented species for each of the five conceptual phyla of the kingdom, their intraspecific variability, and the number of sequences	
*Fusarium solani (Ascomycota)*	3.1%, 542
*Thanatephorus cucumeris (Basidiomycota)*	15.7%, 608
*Olpidium brassicae (Chytridiomycota)*	2.0%, 18
*Glomus intraradices (Glomeromycota)*	8.7%, 92
*Rhizopus oryzae (Zygomycota)*	0.9%, 143
Correlation coefficient for variability between *ITS1* and *ITS2*	0.87 (p-value less than 10^−16^)
Percentage of species where *ITS2* is more variable than *ITS1*	34%
Percentage of species where *ITS1* and *ITS2* differ in variability by less than 0.5%	91%
Percentage of species with either *ITS1* or *ITS2* fully conserved and the other one at least 0.25% variable	22%
Percentage of species with fully conserved *ITS* region	22%
Percentage of species with intraspecific variability ≤3%	75% (*ITS1*), 77% (*ITS2*), 80% (*ITS1*, *5.8S*, *ITS2*)
Percentage of species where the intraspecific variability of *5.8S* is ≤0.5%	80%

**Table 2:** Intraspecific *ITS* variability of select species from each of the five conceptual phyla of the kingdom *Fungi*. Taxon selection was influenced by scheduled, ongoing, and completed genome projects.

Taxonomic affiliation	Sequences	Intraspecific ITS variability
***Ascomycota***		
*Aspergillus fumigatus*	43	0.2%
*Candida albicans*	56	0.2%
*Fusarium solani*	542	3.1%
*Saccharomyces cerevisiae*	145	0.8%
*Xanthoria parietina*	54	0.6%
*Xylaria hypoxylon*	13	24.2%
***Basidiomycota***		
*Amanita muscaria*	45	0.9%
*Boletus edulis*	22	0.3%
*Coprinopsis echinospora*	7	2.6%
*Filobasidiella neoformans*	114	0.0%
*Puccinia graminis*	28	2.4%
*Rhizoctonia bataticola*	6	17.3%
*Ustilago maydis*	5	0.5%
***Chytridiomycota***		
*Olpidium brassicae*	18	2.0%
*Blastocladiella emersonii*	2	2.0%
***Glomeromycota***		
*Archaeospora leptoticha*	62	9.8%
*Glomus intraradices*	92	8.7%
*Glomus mosseae*	84	5.9%
*Paraglomus occultum*	12	19.5%
***Zygomycota***		
*Absidia corymbifera*	9	0.7%
*Endogone pisiformis*	3	2.6%
*Mucor racemosus*	9	8.4%
*Rhizopus oryzae*	143	0.9%
*Zoophthora radicans*	7	1.5%
