# Supplementary figures and images for: Intraspecific ITS Variability in the Kingdom Fungi as Expressed in the International Sequence Databases and Its Implications for Molecular Species Identification
Source: Evol Bioinform Online. 2008 May 26;4:193–201. doi: 10.4137/ebo.s653 (PMC2614188; doi:10.4137/ebo.s653)

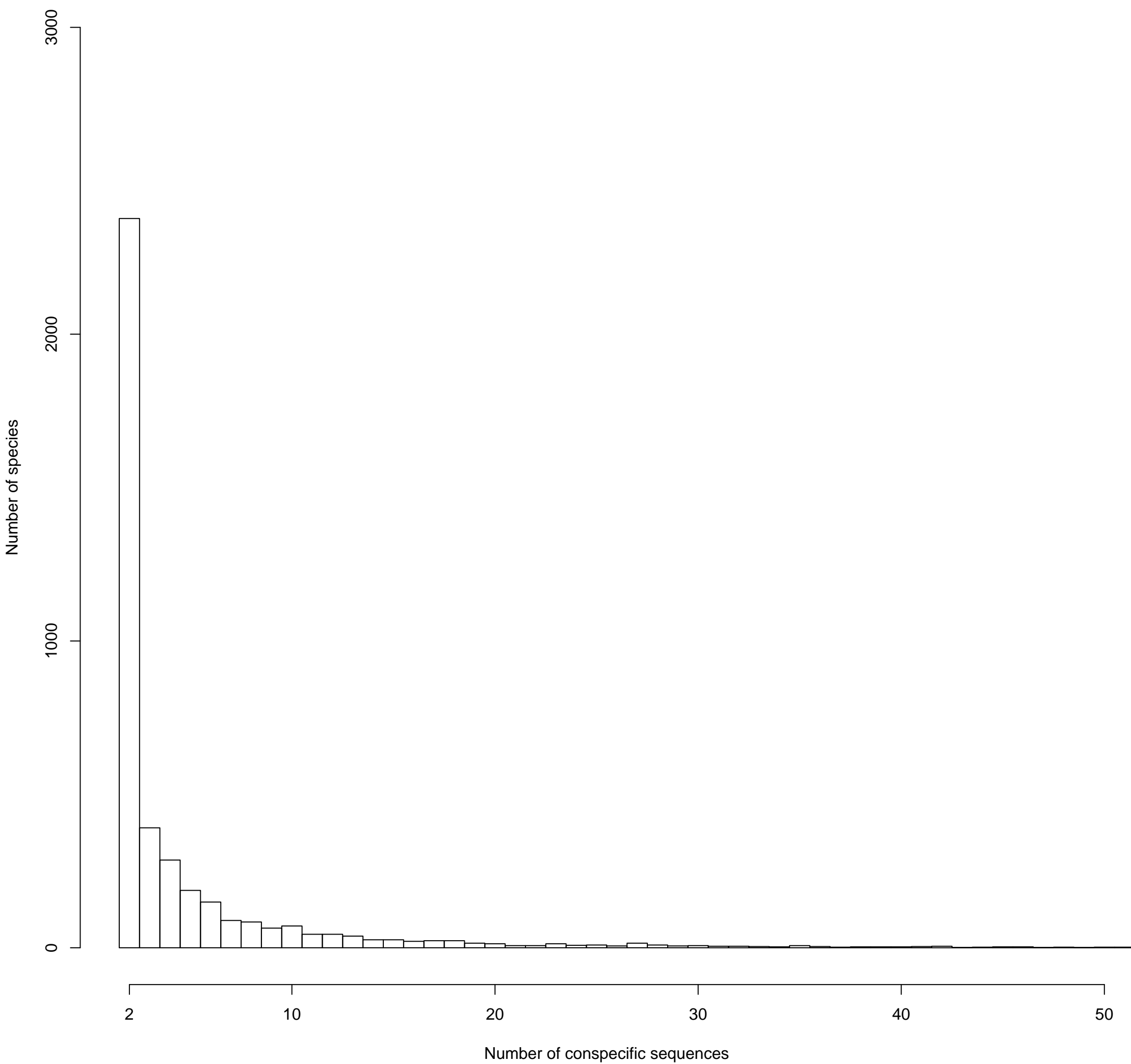

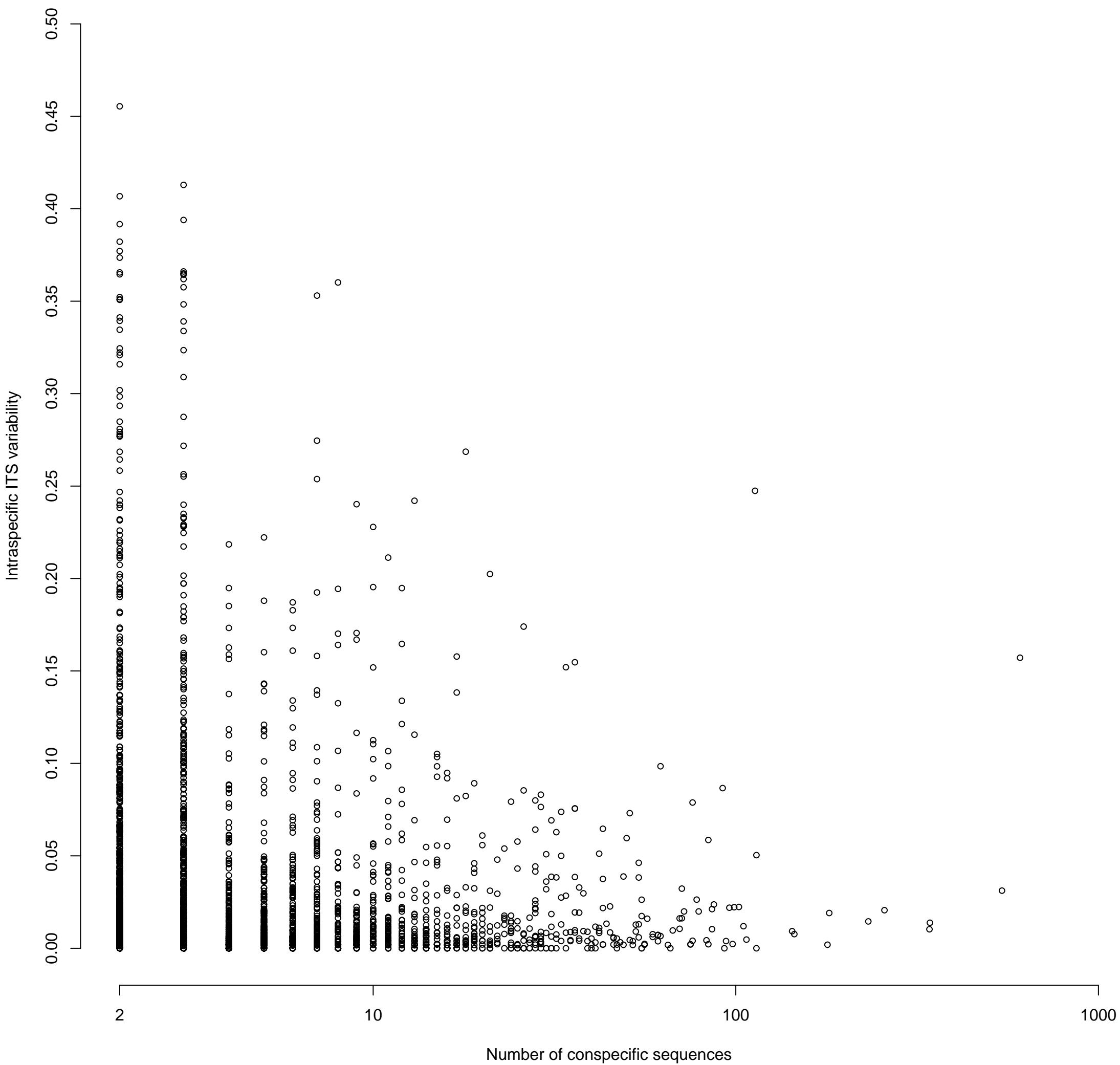

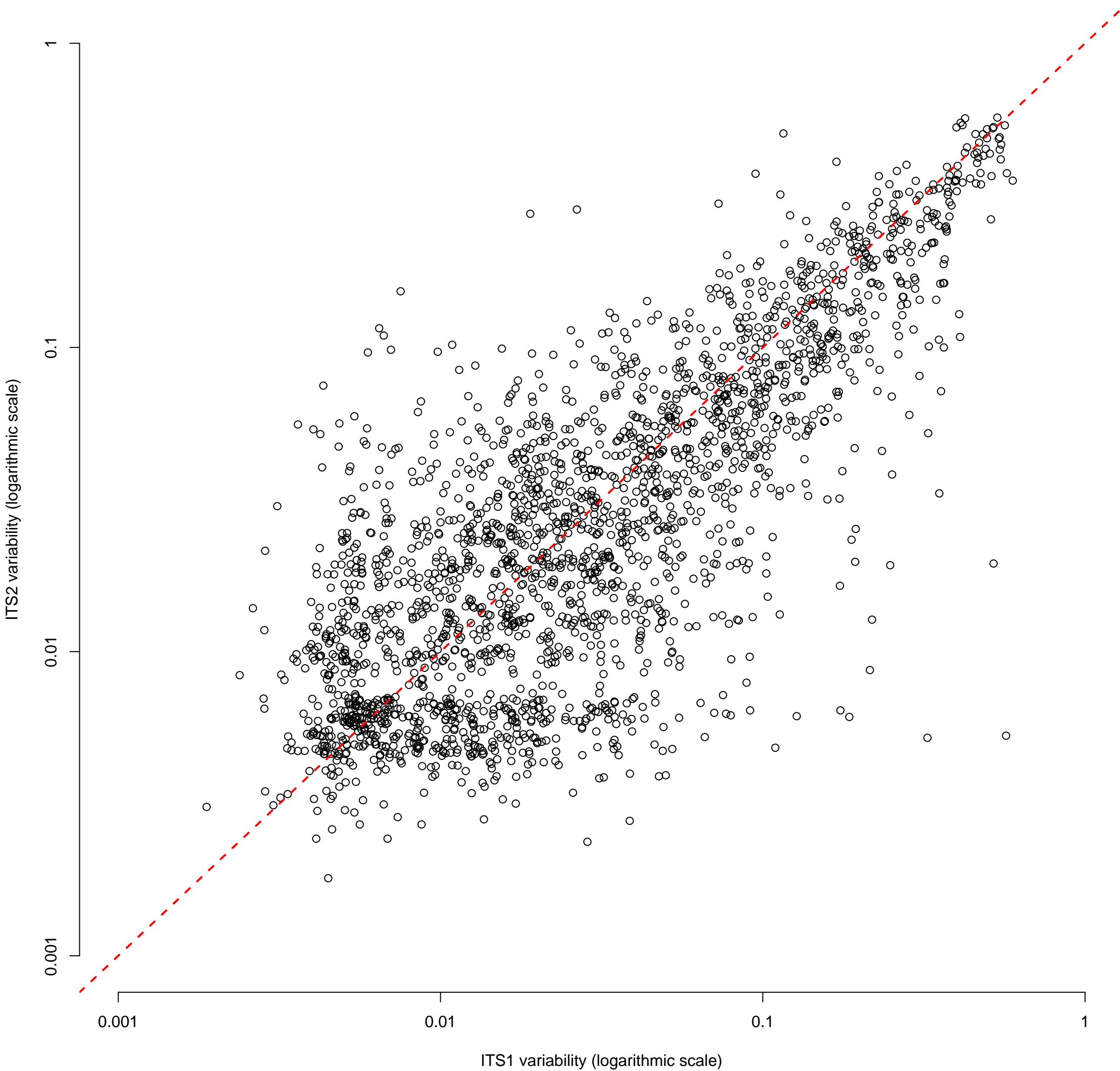

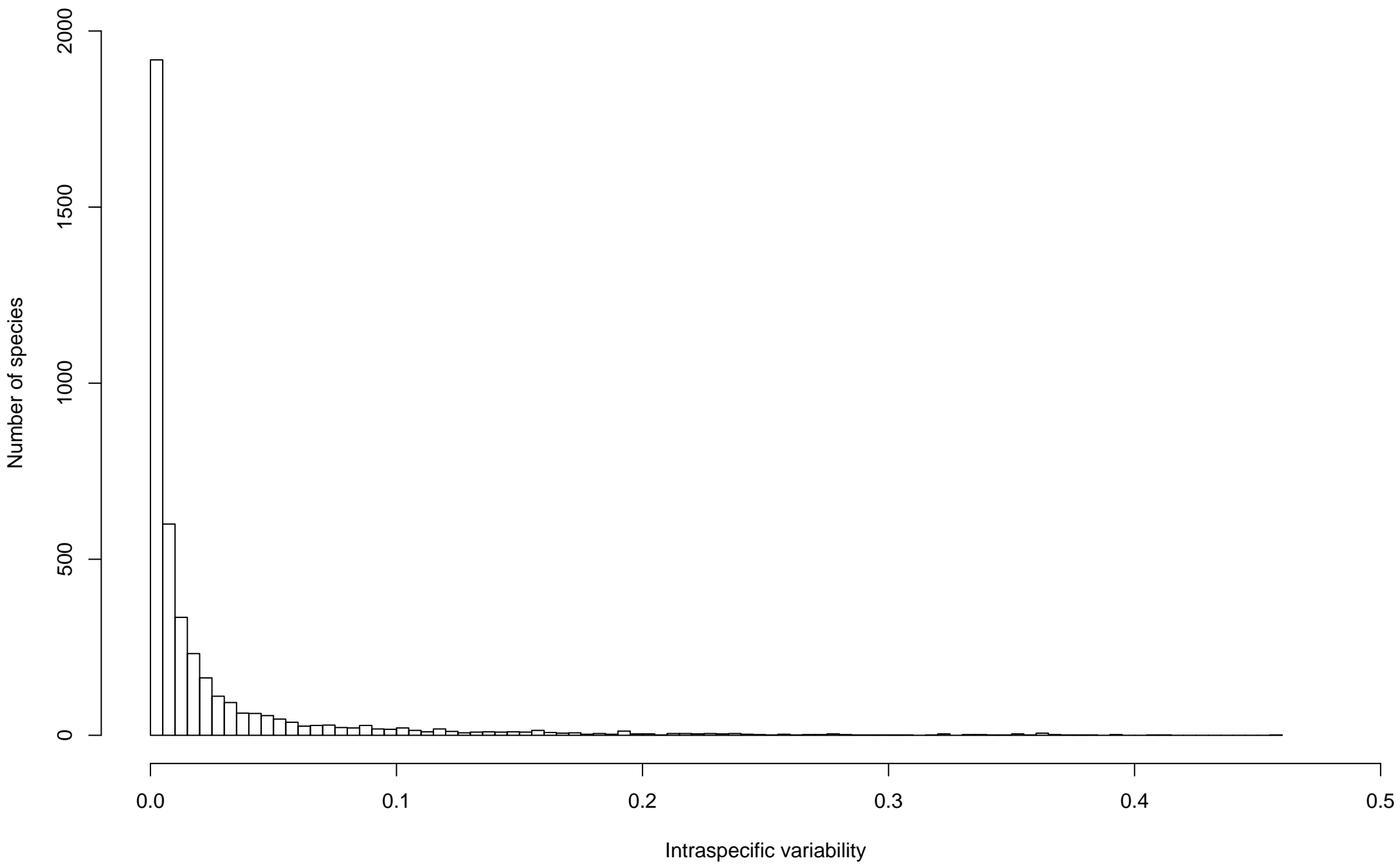

Supplement: Supplementary document 2 — (a) A histogram of the number of fungal ITS sequences per species as included in this study, showing that the majority of species is represented by fewer than five sequences. (b) The number of conspecific sequences plotted against the median intraspecific variability for the species in question, showing a decrease in the uncertainty of the estimates with higher number of sequences. Deviant sequences attain a higher degree of penetration in sparsely sampled species than in more richly sampled ones, where the larger sample sizes lead to estimates of smaller variance. (c) The variability of ITS1 (x axis) plotted against that of ITS2 (y axis) on a logarithmic scale. The correlation coefficient is 0.87 (p-value <10−16). (d) A histogram of the number of species in the study with an intraspecific variability in the ranges indexed, showing the asymmetric, long-tailed distribution of intraspecific variability. Jointly with (a), the histogram gives a good overview of the present state of ITS-borne sampling of fungi. [file ebo-4-193-s2.pdf]
